# Impact of distant peptide substrate residues on enzymatic activity of SlyD

**DOI:** 10.1007/s00018-022-04179-4

**Published:** 2022-02-19

**Authors:** Samuel Pazicky, Anna-Leoni A. Werle, Jian Lei, Christian Löw, Ulrich Weininger

**Affiliations:** 1 Centre for Structural Systems Biology (CSSB), Notkestrasse 85, 22607 Hamburg, Germany; 2 Molecular Biology Laboratory (EMBL), Hamburg Unit C/O Deutsches Elektronen Synchrotron (DESY), Notkestrasse 85, 22607 Hamburg, Germany; 3 Institute of Physics, Biophysics, Martin-Luther-University Halle-Wittenberg, 06120 Halle (Saale), Germany

**Keywords:** Prolyl isomerisation, Enzymatic activity, ITC, NMR, Allosteric regulation

## Abstract

Peptidyl-prolyl isomerases (PPIases) catalyze intrinsically slow and often rate-limiting isomerization of prolyl-peptide bonds in unfolded or partially folded proteins, thereby speeding up the folding process and preventing misfolding. They often possess binding and chaperone domains in addition to the domain carrying the isomerization activity. Although generally, their substrates display no identity in their amino acid sequence upstream and downstream of the proline with 20 possibilities for each residue, PPIases are efficient enzymes. SlyD is a highly efficient PPIase consisting of an isomerase domain and an additional chaperone domain. The binding of peptide substrates to SlyD and its enzymatic activity depend to some extend on the proline-proximal residues, however, the impact of proline-distant residues has not been investigated so far. Here, we introduce a label-free NMR-based method to measure SlyD activity on different peptide substrates and analysed the data in the context of obtained binding affinities and several co-crystal structures. We show that especially charged and aromatic residues up to eight positions downstream and three positions upstream of the proline and outside the canonical region of similar conformations affect the activity and binding, although they rarely display distinct conformations in our crystal structures. We hypothesize that these positions primarily influence the association reaction. In the absence of the chaperone domain the isomerase activity strongly correlates with substrate affinity, whereas additional factors play a role in its presence. The mutual orientation of isomerase and chaperone domains depends on the presence of substrates in both binding sites, implying allosteric regulation of enzymatic activity.

**Supplementary Information:**

The online version contains supplementary material available at 10.1007/s00018-022-04179-4.

## Introduction

Cells possess a plethora of enzymes that facilitate the folding of proteins into their native state to reduce transient accumulation of misfolded proteins [1, 2]. One of the rate-limiting steps in the folding process of proteins to its native state is the isomerization of prolyl-peptide bonds, which typically occurs on the timescale from seconds to hours [3]. This isomerization is catalysed by peptidyl-prolyl isomerases (PPIases) [4–8], which can be divided in three classes: FK506 binding proteins (FKBPs) [9], cyclophilins [5] and parvulins [10]. Ubiquitously expressed FKBPs can either exist as individual proteins or constitute a domain in a larger protein. Often, FKBPs are linked to an additional chaperone domain that can increase their PPIase activity by up to 200-fold [11–13]. The reported catalytic efficiencies of such enzymes (*k*_cat_/*K*_m_) reach 10^8^ M^−1^ s^−1^ [14], which classifies them as superefficient enzymes with turn-over rates limited by diffusional association [15]. This is specifically intriguing for PPIases that possess highly promiscuous binding sites that interact with a large variety of different peptides and proteins [14, 16–18].

**Fig. 1:**
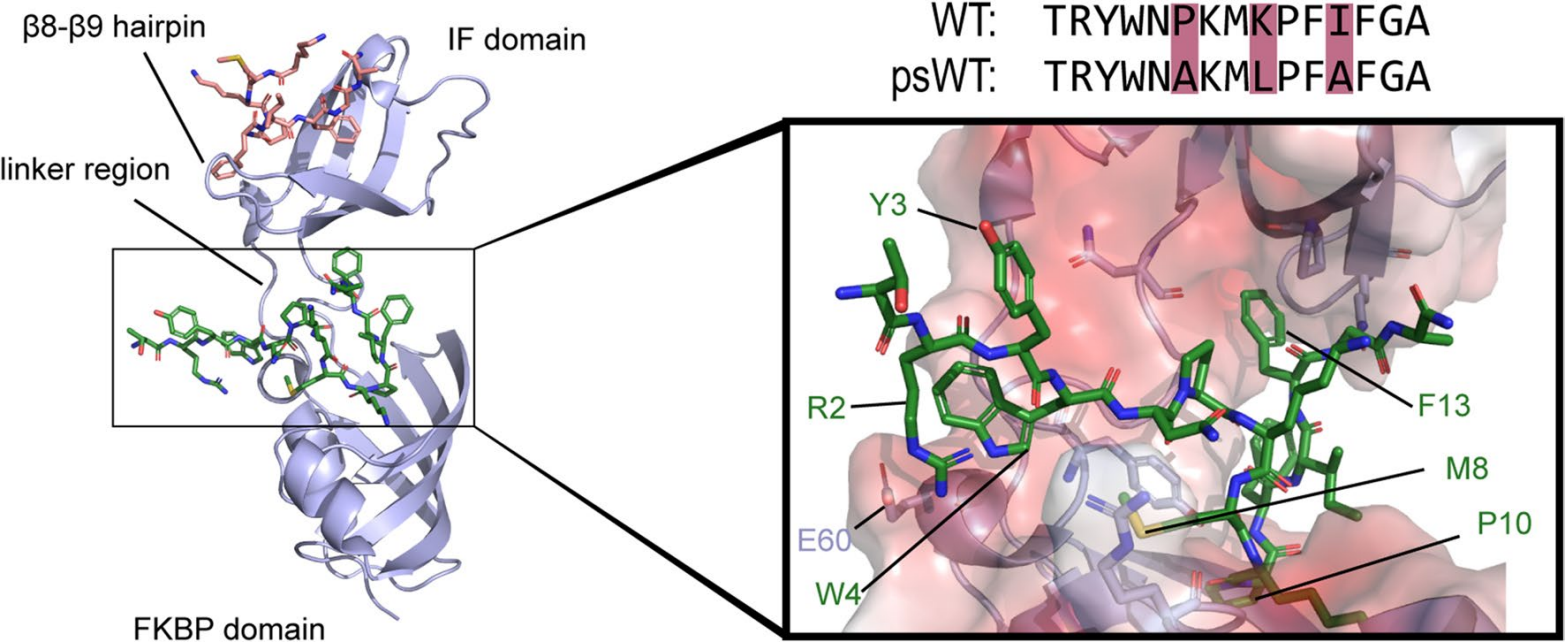
Peptide binding of SlyD. Structure of SlyD^WT^(4odl.pdb) with a peptide derived from ribosomal S2 protein bound in the FKBP binding site (substrate in green) and in the IF binding site (substrate in pink).The sequences of the S2-derived wild-type peptide (WT) and derived pseudo-wild-type (psWT) peptide used in this study with the exchanged residues are shown. Zoom: The peptide substrate bound in the FKBP binding site with interacting residues labelled

An example of such a protein is SlyD (Sensitive to Lysis D), which consists of an FKBP type PPIase domain and a chaperone domain, called IF (insert-in-flap) domain, that has been shown to exhibit chaperone activity [19–21]. The PPIase domain maintains a reduced function even in the absence of the chaperone domain. Each of these domains contains one binding site for unfolded proteins or peptides (Fig. 1). The PPIase and chaperone domains are expected to function independent of each other, as shown by NMR structure analysis and by various crystal structures, where both domains showed a certain degree of variability in their orientations [14, 19, 21]. However, it has been suggested that inter-domain cross-talk plays a role in the function of SlyD, despite the mutual plasticity of the two domains. It was shown by smFRET analysis [22] that the binding of substrates does not alter the dynamics between the two domains. On the other hand, NMR dynamic experiments proposed that binding of unfolded proteins in the IF domain triggers structural changes in the FKBP domain [23]. To this date it is not clear whether an inter-domain cross-talk exists and the chaperone domain actively delivers substrates to the active site of the PPIase domain, or if the pure presence of the chaperone domain in direct neighbourhood is the cause for the increased activity.

The activity of PPIases has been studied by multiple methods [24–31], including variants of assays using tetrapeptides with a C-terminal fluorescent reporter, tetrapeptide-4-nitroanilide, and NMR measurements using ^13^C-labeled peptides. Thorough studies were performed on the importance of the substrate residue in *i* − 1 and *i* + 1 position (preceding and following the proline residue), revealing that aromatic residues trigger the highest PPIase activity, followed by arginine and small hydrophobic amino acids. While these approaches enabled identification of proline-neighbouring residues that have an impact on PPIase activity, they limit the measurements to only short peptides [32, 33]. Longer peptides (15 residues), which can be considered as more natural substrates, have previously been shown to be far better substrates for *Thermus thermophilus* SlyD (further referred to as SlyD) [14]. Moreover, the crystal structures of SlyD bound to this 15-residue-long peptide revealed potential interactions with the proline-distant residues (Fig. 1). Additionally, the peptides bound in different conformations to the chaperone domain binding site and in one case, even an inverted orientation relative to the FKBP binding site was observed.

Here, we set out to investigate how substrate residues distant from the proline influence the isomerase activity of SlyD. We studied the activity with a label-free NMR-based method suitable for longer substrates as well as the interactions using ITC and structural features from high-resolution crystal structures. Residues eight positions downstream and three positions upstream of the proline residues impact enzymatic activity and binding, in particular their charged and aromatic variants. These residues are often not conformationally restricted and either display different orientations/conformations in crystal structures or are not resolved at all. We hypothesize that these positions are most likely important for the association reaction and necessarily present in the formed complex. In the absence of the chaperone domain, the SlyD activity correlates linearly with substrate binding affinity, whereas in its presence, additional factors contribute to the enzymatic activity. First, the chaperone domain can contribute to the directionality of the substrate binding in the PPIase domain, as is apparent from our crystal structures of a peptide substrate bound in a reverse direction in the absence of chaperon domain. Second, our crystal structures show that the absence or presence of the substrates in the PPIase and chaperon binding sites affect the mutual orientation of these domains, demonstrating possible allosteric regulation that influences the accessibility of the PPIase domain for substrate binding.

## Materials and methods

### Protein samples

The SlyD constructs were expressed and purified as described previously [19]. Briefly, the proteins were expressed in *E. coli* overnight at 18 °C upon induction with 0.2 mM IPTG. The harvested cell pellet was lysed and the lysate supernatant was bound to Ni NTA beads. The proteins were unfolded on the beads by washing with a buffer containing 6 M guanidine chloride, refolded by decreasing concentration of guanidine chloride and eluted with an increased concentration of imidazole. The concentrated eluate was incubated with 10 mM EDTA and further purified by size exclusion chromatography with Superdex 75 column in 20 mM HEPES (pH 7.5) and 100 mM NaCl. The SlyD-containing peak fractions were concentrated and flash-frozen in liquid nitrogen.

### Enzyme activity by NMR lineshape analysis

^1^H NMR spectra of 100 µM peptide samples in 20 mM HEPES, 100 mM NaCl, pH 7.5 were acquired on a Bruker Avance III NMR-spectrometer at 25 °C and different concentrations of enzyme (0 to 6 µM). Samples contained 10% (v/v) D_2_O and 0.5 mM TCEP. Spectra have been corrected by subtracting identically measured and processed spectra of enzyme only samples, in order to rule out any influence of signals from the enzymes. Spectra were processed using Topspin (Bruker, Inc) and analyzed in MATLAB (mathworks). By lineshape analysis of one methyl group (I) of the leucin residue (*i* − 1 of the proline), which displays different chemical shifts for the *cis* and the *trans* form, apparent exchange rates between the *cis* and the *trans* state were derived and used for obtaining k_cat_/K_M_ values by linear regression vs. enzyme concentrations [14, 26]. Intrinsic line widths (including possible inhomogeneities) under each conditions have been obtained initially by fitting the second methyl group of the leucine residue (II), which does not display differences between the *cis* and *trans* form, and therefore experiences no broadening from chemical exchange. The obtained relative line broadening of the *cis* and *trans* signals of methyl group I*,* is caused by chemical exchange between the two forms and depends on the population of the *cis* and *trans* forms, their chemical shift difference (which are both known from experiments without enzyme) and the apparent exchange rate which was derived from the fit. Errors in the fitted parameters were estimated using Monte-Carlo simulations [34]; the reported errors correspond to one standard deviation.

### Michaelis–Menten formalism

The Michaelis–Menten model is schematically described as

1 $$\begin{array}{*{20}c} {E + S \begin{array}{*{20}c} {\begin{array}{*{20}c} {k_{on} } \\ \rightharpoonup \\ \end{array} } \\ {\begin{array}{*{20}c} \leftharpoondown \\ {k_{off} } \\ \end{array} } \\ \end{array} ES \begin{array}{*{20}c} {\begin{array}{*{20}c} {k_{cat} } \\ \rightharpoonup \\ \end{array} } \\ {} \\ \end{array} E + P } \\ \end{array}$$

where *k*_*on*_ and *k*_*off*_ are the rate constants at which the enzyme binds and releases the substrate and *k*_*cat*_ is the rate constant of catalysis, E is the enzyme, S the substrate, ES the enzyme substrate complex and P the product.

The Michaelis constant (*K*_*M*_) is given as

2 $$\begin{array}{*{20}c} {K_{{\text{M}}} = \frac{{k_{{{\text{off}}}} + k_{{{\text{cat}}}} }}{{k_{{{\text{on}}}} }}} \\ \end{array} ,$$

is defined as the substrate concentration in which have the enzymatic velocity attains half its maximal value. For substrate concentrations substantially below *K*_*M*_ one obtains the enzymatic activity

3 $$\frac{{k_{{{\text{cat}}}} }}{{K_{{\text{M}}} }} = { }\frac{{k_{{{\text{on}}}} \times k_{{{\text{cat}}}} }}{{k_{{{\text{off}}}} + k_{{{\text{cat}}}} }},$$

and substantially above *K*_*M*_ one obtains *k*_*ca*t_; at given enzyme and substrate concentrations. If *k*_off_ is substantially higher than *k*_*cat*_ Eq. 3 simplifies to

$$\frac{{k_{{{\text{cat}}}} }}{{K_{{\text{M}}} }} = { }\frac{{k_{{{\text{on}}}} \times k_{{{\text{cat}}}} }}{{k_{{{\text{off}}}} }} = { }\frac{{k_{{{\text{cat}}}} }}{{K_{{\text{D}}} }}.$$

Here the enzyme substrate complex is formed multiple times before catalysis occurs. The Michaelis constant can be interpreted as the dissociation constant K_D_. This resemble the EX2 exchange regime in amide exchange experiments. Increasing *k*_ca*t*_ or the binding strength (lower *K*_*D*_) increases the enzymatic efficiency. If on the other hand *k*_cat_ is substantially higher than *k*_off_ Eq. 3 simplifies to

5 $$\frac{{k_{{{\text{cat}}}} }}{{K_{{\text{M}}} }} = k_{{{\text{on}}}} .$$

Here every formation of the enzyme substrate complex results in catalysis. This resemble the EX1 exchange regime in amide exchange experiments. Only increasing the association of the substrate (*k*_on_) increases the enzymatic efficiency. Catalysis becomes association or diffusion limited. In SlyD *k*_off_ can estimated assuming diffusion limited association *k*_on_ of 10^8^ M^−1^s^−1^ and dissociation constants of 50 µM or below (Table 1) to 5000 s^−1^ or less, which is far below the determined *k*_cat_ of (740 000 ± 140 000) s^−1^ [14].

**Table 1:** Thermodynamic constants of substrate binding to SlyD^WT^ and SlyDΔIF and their respective enzymatic activities

	SlyD^WT^					SlyDΔIF		
Mutant	K_D, IF_ (µM)	ΔH_IF_ (kcal/mol)	K_D, FKBP_ (µM)	ΔH_FKBP_ (kcal/mol)	k_cat_/K_M_ (µM^−1^ s^−1^)	K_D_ (µM)	ΔH (kcal/mol)	k_cat_/K_M_ (µM^−1^ s^−1^)
psWT	0.22 ± 0.01	− 15.4 ± 0.1	3.5 ± 0.1	− 6.4 ± 0.2	4.9 ± 0.3	15.6 ± 0.2	− 12 ± 0.1	2.2 ± 0.3
R2A	n.d	n.d	n.d	n.d	5.1 ± 0.3	n.d	n.d	1.3 ± 0.2
Y3A	0.72 ± 0.1	− 13.1 ± 0.1	17.3 ± 1.3	− 11.3 ± 0.5	3.9 ± 0.4	51.5 ± 8.6	− 9.3 ± 1.9	2.5 ± 0.3
W4A	0.75 ± 0.06	− 15.6 ± 0.1	8.4 ± 0.4	− 6.6 ± 0.7	2.0 ± 0.2	30.8. ± 4.0	− 6.6 ± 0.7	1.1 ± 0.2
W4E	0.86 ± 0.02	− 15.2 ± 0.1	13.9 ± 0.9	− 5.1 ± 0.7	4.4 ± 0.2	19.6. ± 3.4	− 3.8 ± 0.5	0.9 ± 0.2
W4K	0.21 ± 0.01	− 15.5 ± 0.1	3.2 ± 0.3	− 3.1 ± 0.4	2.2 ± 0.3	12.6 ± 0.8	− 4.4 ± 0.2	3.5 ± 0.7
M8A	0.24 ± 0.01	− 18.5 ± 0.1	6.0 ± 0.1	− 9.3 ± 0.2	7.7 ± 0.9	19.4 ± 1.0	− 10 ± 0.3	3.1 ± 0.1
F13A	0.38 ± 0.02	− 17.6 ± 0.1	6.4 ± 0.3	− 6.4 ± 0.4	3.1 ± 0.1	22.7 ± 0.6	− 15 ± 0.3	0.4 ± 0.1
F13E	1.15 ± 0.01	− 20.8 ± 0.2	10.4 ± 0.5	− 5.0 ± 0.8	7.0 ± 0.6	36.1 ± 0.8	− 13 ± 0.2	0.1 ± 0.1
F13K	0.11 ± 0.02	− 18.1 ± 0.1	5.2 ± 0.2	− 5.2 ± 0.2	3.4 ± 0.1	20.0 ± 0.7	− 10 ± 0.3	2.3 ± 0.3
G14M	0.10 ± 0.01	− 16.6 ± 0.2	2.2 ± 0.1	− 8.2 ± 0.3	n.d	8.3 ± 0.5	− 11 ± 0.2	n.d
A15L	0.16 ± 0.02	− 14.3 ± 0.2	4.1 ± 0.1	− 6.5 ± 0.4	n.d	14.8 ± 0.2	− 10 ± 0.1	n.d

### Isothermal titration calorimetry (ITC)

ITC was measured using MicroCal VP-ITC calorimeter (Malvern) and analysed with MicroCal ITC-ORIGIN Analysis Software. First 1 × 3 µl and then 42 × 6.5 µl of 1.2 mM peptides dissolved in 20 mM HEPES (pH 7.5) and 100 mM NaCl were injected every 240 s from the syringe into the cell containing 50 µM SlyD at 25 °C. The resulting isotherms were fitted with one binding site model in case of SlyDΔIF construct and with two binding site model in case of the wild-type SlyD.

### Correlation analysis

Spearman’s rank correlation coefficient was calculated in R version 3.6.3 [35] using package Hmisc [36] with the exclusion of outliers identified by bagplots using the R package aplpack [37]. The PCA was performed using the R package factoextra [38].

### X-ray crystallography

The peptide substrates were directly dissolved in the purified protein concentrated to 60 mg/ml to yield a molar ratio of approximately 3:1 (peptide:protein), respectively, and the sitting drop crystallization screens were set up at 19 °C. The individual peptide:protein complexes were crystallized after mixing in 1:1 ratio (final volume 300 nl) with the following crystallization conditions: SlyD^WT^ + W4A in 0.1 M sodium cacodylate (pH 6.5), 40% MPD, 5% PEG8000; SlyD^WT^ + W4K 0.1 M bicine (pH 9), 10% PEG 20,000, 2% 1-4dioxane; SlyD^WT^ + M8A and SlyD^WT^ + psWT in 0.1 M sodium cacodylate (pH 6.5), 50% PEG 200; and SlyDΔIF + M8A in 0.1 M Bis Tris (pH 5.5), 20% PEG 3350. The crystals appeared and stopped growing after 3–7 days, and were subsequently flash-frozen in liquid nitrogen.

### Data collection and structure determination

The diffraction data were collected at the P13 EMBL beamline of the PETRA III storage ring (c/o DESY, Hamburg, Germany) at 0.966 Å wavelength and 100 °K temperature using a Pilatus 6 M detector (DECTRIS). The raw data were processed with XDS [39] merged with Aimless [40] and the phases were obtained by molecular replacement with Phaser [41], using separated domains (FKBP domain residues 1–66 + 125–150 and IF domain residues 70–117) from previously determined SlyD structure (pdb 3cgm [19]) as a search model. In all cases, the models were further built and refined in several cycles using PHENIX [42], Refmac [43] and Coot [44]. Data collection and refinement statistics are summarized in Table 2. PyMOL was used to generate the figures and root mean square deviations (RMSDs). PDBePISA [45] was used to characterize the intermolecular interfaces. The atomic coordinates and the structure factors have been deposited in the PDB with accession numbers 7oxg, 7oxh, 7oxi, 7oxjand 7oxk.

**Table 2:** X-ray data collection and refinement statistics

	SlyD^WT^ + psWT	SlyD^WT^ + M8A	SlyD^WT^ + W4A	SlyD^WT^ + W4K	SlyDΔIF + M8A
Data collection					
Beamline	PETRA III P14	PETRA III P14	PETRA III P13	PETRA III P13	PETRA III P13
Wavelength	0.9763	0.9763	1.0332	1.0332	0.9763
Space group	P 31 2 1	P 31 2 1	P 64 2 2	P 64 2 2	P 1 21 1
Cell dimensions					
*a*, *b*, *c* (Å)	49.23, 49.23, 131.2	49.28, 49.28, 130.7	108.9, 108.9, 91.06	109.4, 109.4, 93.64	34.68, 82.53, 42.27
α, β, γ (°)	90, 90, 120	90, 90, 120	90, 90, 120	90, 90, 120	90, 110.5, 90
Resolution (Å)	40.54–1.70 (1.76–1.70)	42.68–1.85 (1.92–1.85)	41.86–2.60 (2.70–2.60)	47.36–2.80 (2.90–2.80)	39.59–2.00 (2.07–2.00)
R_merge_	0.0360 (0.697)	0.0417 (1.040)	0.0567 (2.105)	0.0991 (3.325)	0.1084 (0.947)
I / σI	37.59 (4.02)	32.99 (3.30)	30.51 (1.43)	19.68 (0.95)	9.89 (1.69)
Completeness (%)	99.8 (99.2)	99.9 (99.6)	99.9 (99.4)	99.9 (99.6)	99.4 (99.4)
Total no. reflections	300,226 (28,778)	238,194 (24,016)	188,806 (17,680)	157,669 (15,725)	72,136 (7333)
Wilson B-factor	33.64	38.36	95.41	101.86	31.43
Multiplicity	14.3 (14.1)	14.6 (15.1)	18.5 (17.9)	18.4 (19.1)	4.8 (5.0)
Refinement					
Resolution (Å)	1.70	1.85	2.60	2.80	2.00
No. reflections	300,226	238,194	188,806	157,669	72,136
R_work_ / R_free_	0.203/0.239	0.199/0.224	0.217/0.245	0.217/0.235	0.202/0.247
No. atoms	1413	1389	1362	1368	1849
Protein	1292	1267	1357	1365	1777
Ligands	46	36	1	n.a	7
Solvents	75	86	4	3	65
B-factors	53.83	58.94	119.85	122.28	45.43
Protein	53.63	59.07	119.98	122.32	45.42
Ligands	57.78	50.79	91.58	n.a	54.61
Solvent	54.78	60.49	85.89	105.15	44.73
R.m.s. deviations					
Bond lengths (Å)	0.007	0.009	0.013	0.006	0.005
Angles (°)	0.96	1.11	1.85	1.05	0.93
Ramachandran					
Favoured (%)	98.15	98.11	98.22	98.22	100
Outliers (%)	0	0	0	0.7	0
Clashscore	4.95	2.76	20.11	8.99	3.14
PDB accession number	7oxh	7oxh	7oxi	7oxk	7oxg

## Results

### NMR based (label-free) prolyl isomerase activity assay for longer peptide substrates

**Fig. 2:**
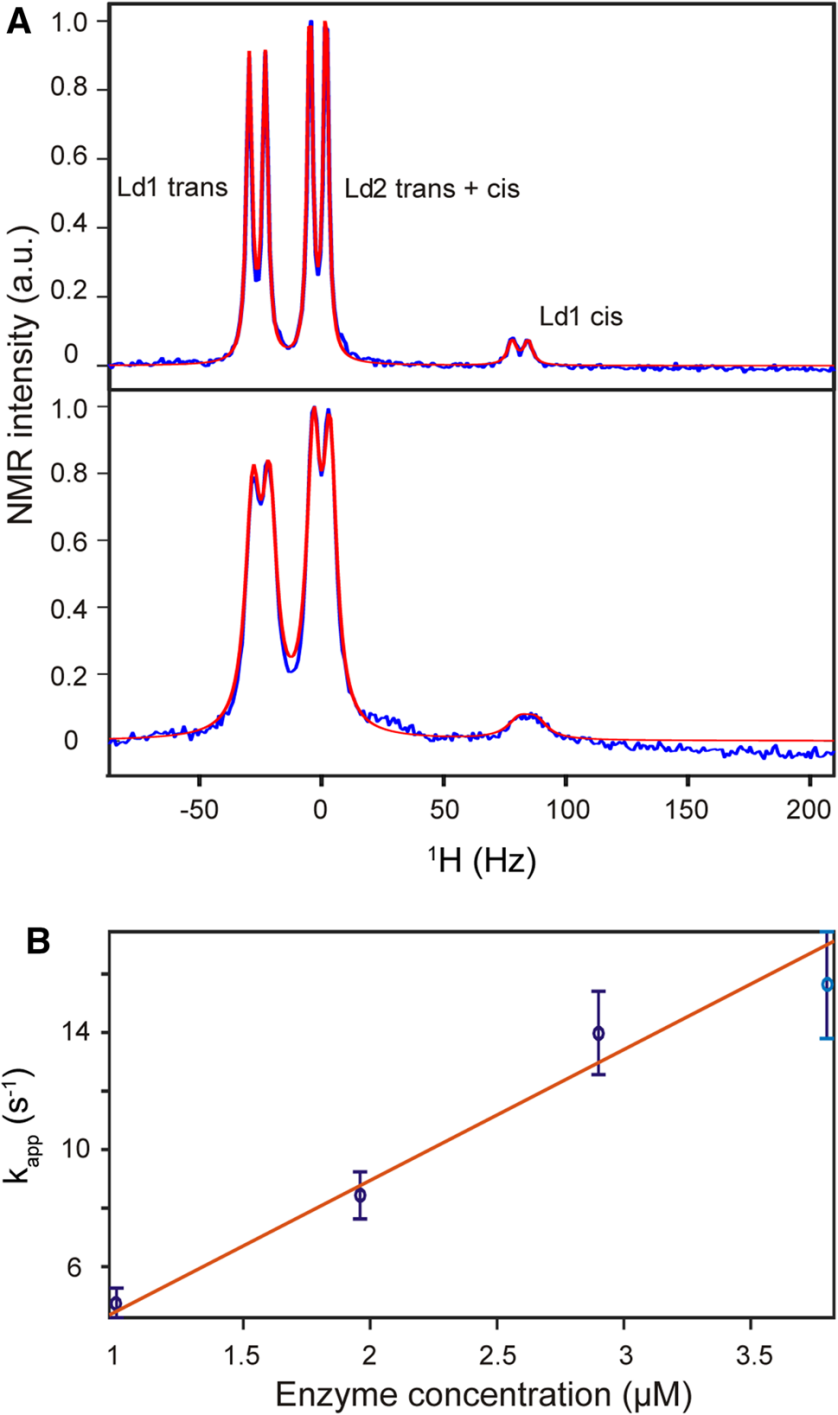
NMR-based label-free method for measuring SlyD activity. **A** Region of Leu methyl groups in a ^1^H NMR spectrum of 100 µM psWT peptide and 1 µM SlyD (up) and 4 µM SlyD (down). Experimental spectra are shown in blue and fitted spectra are shown in red. **B** Derived exchange rates are plotted against enzyme concentration and fitted linearly in order to derive *k*_cat_*/K*_M_

Tetrapeptides terminally fused with fluorophores sensitive to proline isomerization have been routinely used to study the activity of peptidyl-prolyl *cis*–*trans* isomerases, such as SlyD [11]. However, this approach is not applicable for longer peptide substrates, as a conformational change of the proline residue does not translate into a change in the terminal fluorophore because of the increased distance involved. Studying longer peptides usually requires 2D NMR spectroscopy, often in combination with ^13^C labeling [14, 30], in order to detect separate signals for the *cis* and *trans* state. Here, we developed a 1D ^1^H NMR method for longer peptides that does not require a fluorophore, chemical modification or ^13^C labeling of the substrate peptide. This approach, which was initially developed for chemically modified tetrapeptides [26], relies only on the interaction of a leucine residue upstream of the proline (*i* − 1) and a phenylalanine residue downstream of the proline (*i* + 1). The different orientations of the leucine and phenylalanine residues in *cis* and *trans* proline conformations give rise to different signals for one of the leucine methyl groups (I), while the other methyl group (II) is mostly unaffected (Fig. 2A) [14, 26]. At the same time, the impact of the proline *cis/trans* conformation on the leucine methyl groups is not dependent on the length of the peptide. The only requirement for the design of the peptide substrate is the absence of additional leucine, isoleucine or valine residues that could interfere with the NMR signals of interest. The linewidth of the signals is affected by the enzymatic turnover rate, which itself is dependent on the enzyme concentration (Fig. 2A). The enzymatic activity (*k*_ca*t*_*/K*_M_) is then determined by the linear regression of the determined rate at various enzyme concentrations at a fixed substrate concentration below *K*_M_ (Fig. 2B and SI Fig. 1).

### Residues distant from the Proline residue influence enzymatic activity

**Fig. 3:**
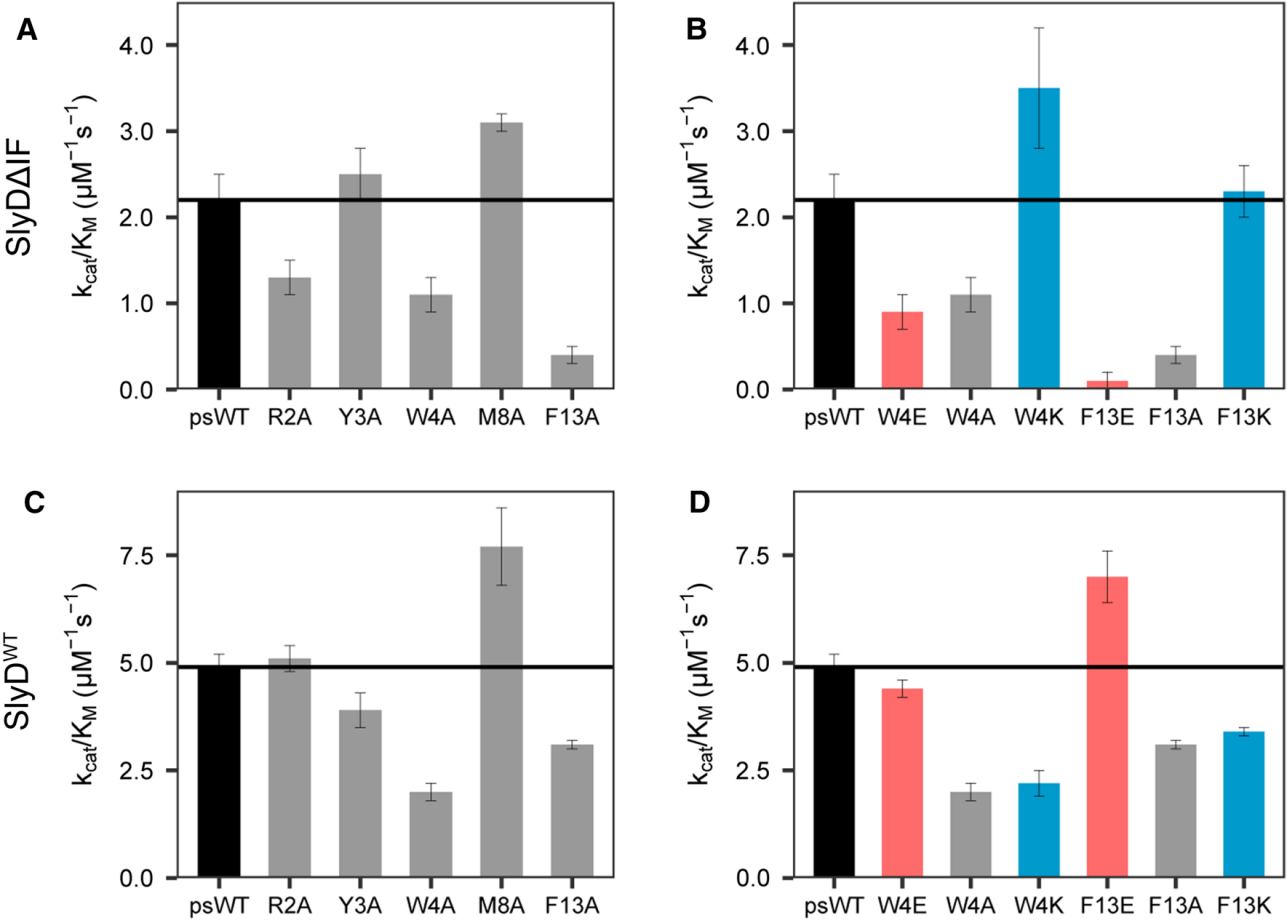
Enzymatic activity (*k*_cat_*/K*_M_) of SlyDΔIF and SlyD^WT^ with different mutants of the peptide substrate. **A** Enzymatic activity of SlyDΔIF on alanine peptide mutants. **B** Enzymatic activity of SlyDΔIF on charged mutants of peptide residues W4 and F13. **C** Enzymatic activity of SlyD^WT^ on alanine peptide mutants. **D** Enzymatic activity of SlyD^WT^ on charged mutants of peptide residues W4 and F13. The alanine mutants are grey, glutamate mutants red, lysine mutants blue and psWT peptide black. The measurements were performed in technical triplicates

We applied our label-free method to explore the impact of proline-distant residues on the enzymatic activity of SlyD. To this end, we used the 15-residue-long peptide derived from the ribosomal protein S2 (Fig. 1), as this peptide displays higher affinity to both SlyD domains and higher turnover rates as compared to tetrapeptides or protein substrates [14]. The original S2 peptide sequence (NH_2_-TRYWNPKMKPFIFGA-COOH) was modified such that the first proline residue was changed to alanine (P6A), a leucine residue was placed upstream of the second proline residue (K9L) and the isoleucine residue was mutated to alanine (I12A), resulting in the pseudo-wild-type (psWT) peptide:NH_2_-TRYWNAKMLPFAFGA-COOH (Fig. 1). We then performed an alanine scan on residues that were shown to interact with SlyD in the previously determined crystal structures (Fig. 1) [14], namely R2, Y3, W4, M8 and F13. We measured the rate of prolyl isomerization in the presence of wild-type SlyD (SlyD^WT^) and a construct lacking the IF chaperone domain (SlyDΔIF) (Fig. 3). For SlyDΔIF, the mutation Y3A does not have an impact on activity as compared to the psWT peptide, while R2Aand W4A lead to a modest, and F13A to a strong decrease in the enzymatic activity (Fig. 3A). In contrast, the activity of SlyDΔIF on the peptide carrying the mutation M8A is increased. In order to further explore the contribution of the aromatic residues W4 and F13 of the peptide substrate on the SlyD activity, we changed them individually to negatively charged glutamate (W4E, F13E) or positively charged lysine (W4K, F13K) residues. For the glutamate variants, we observed a similarly decreased activity as for the alanine mutants, while lysine variants resulted in a similar or even increased activity as the psWT peptide (Fig. 3B). Thus, aromatic and positively charged amino acid residues at positions 4 and 13 (*i *− 6 and *i* + 3 of P10, respectively) increase the enzymatic activity of SlyDΔIF. As the enzymatic activity of very efficient enzymes with high turnover rates (*k*_cat_), such as SlyD, is coupled to the association reaction (*k*_on;_ see materials and methods), we conclude that the observed differences in the activities of the different mutant peptides are most likely caused by different association kinetics of the enzyme substrate complex. The strong impact of aromatic residues, which are mainly of hydrophobic nature and can form various interactions, such as aromatic interaction and cation-π stacking, further supports this conclusion.

Overall, the enzymatic activity of the SlyD^WT^ is higher as compared to that of SlyDΔIF (Fig. 3C, D) due to the presence of the additional chaperone domain. The activity profile for peptides with alanine residues in different positions confirms the importance of W4 and F13 to the enzymatic activity in the full-length context, while removing the methionine side chain in the M8A peptide increases the enzymatic activity. In contrast to SlyDΔIF, the mutation R2A causes no difference in SlyD^WT^ activity as compared to the psWT peptide, while residue Y3 is of minor importance. Upon replacement of W4 and F13 by negatively charged glutamate or positively charged lysine residues, an opposite picture arises as seen for SlyDΔIF (Fig. 3B vs D). SlyD^WT^ is less active on peptides with lysine residues in position 4 and 13, similar to the alanine mutants, while the activity towards glutamate variants is increased or unchanged (Fig. 3D).

Comparing the enzymatic activity of SlyD^WT^ and SlyDΔIF on different peptide substrates highlights the beneficial contribution of the chaperone domain on the isomerization reaction. We did not observe any general correlation between the activities of SlyD^WT^ and SlyDΔIF (*ρ* = − 0.38, *p* = 0.36*,* SI Fig. 2DE). We would assume a correlation, if the chaperone domain simply increases the association rate of the substrate. The absence of such a correlation demonstrates the complex nature of the protein and of the interplay between its two domains. Taken together, the activity data illustrate that the changes in amino acid residues distant from the isomerized proline residue impact SlyD activity.

### The chaperone domain of SlyD increases the substrate affinity to PPIase domain in a linear manner

**Fig. 4:**
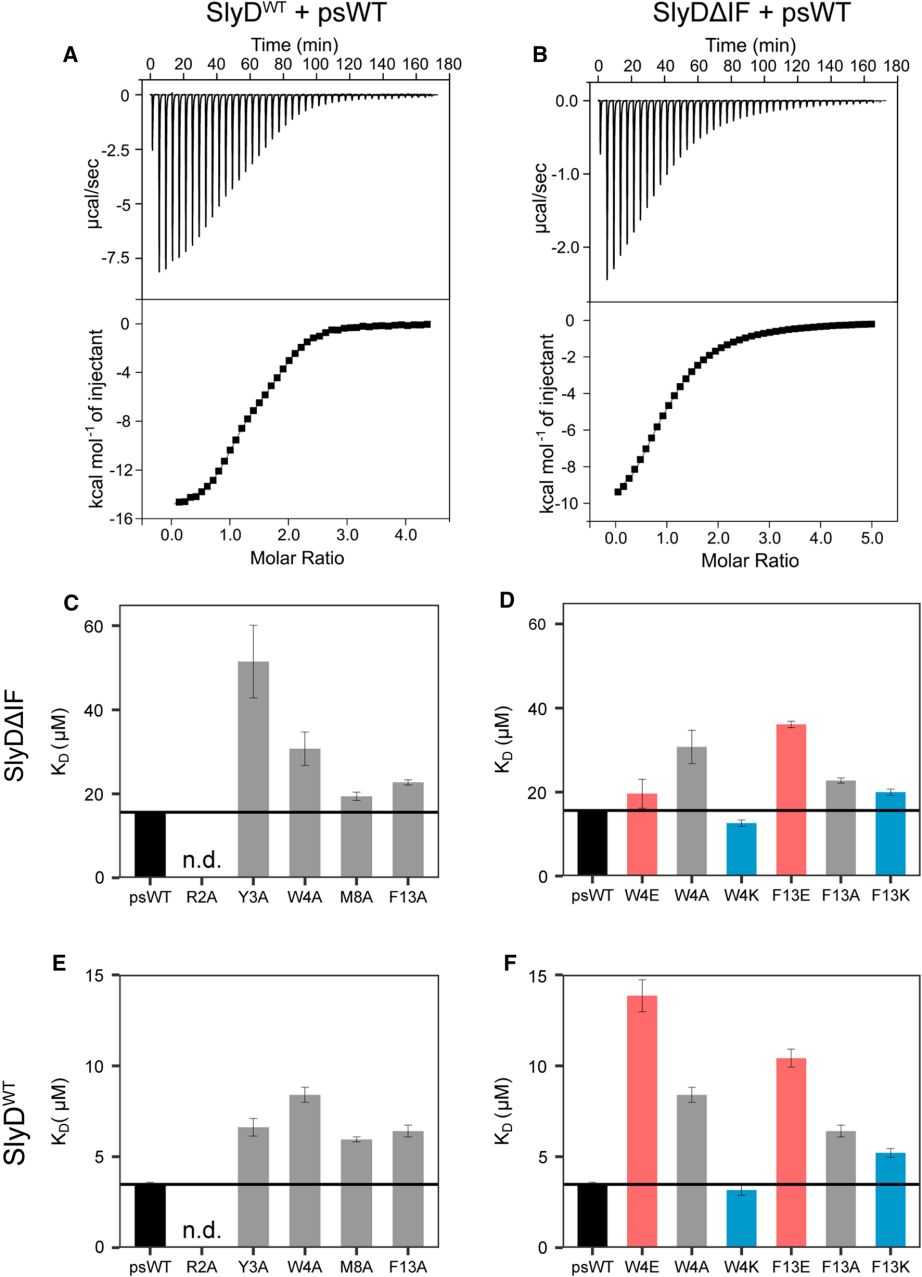
Affinities of substrate peptides to SlyDΔIF and SlyD^WT^ FKBP domain measured by ITC. **A** Example of an ITC measurement with the psWT peptide substrate binding to SlyD^WT^ protein fitted a with two-binding-sites model. **B** Example of an ITC measurement with psWT peptide substrate binding to SlyDΔIF construct that possesses only one binding site. **C** Dissociation constant of SlyDΔIF binding to alanine peptide mutants. **D** Dissociation constant of SlyDΔIF binding to charged mutants of peptide residues W4 and F13. **E** Dissociation constant of SlyD^WT^ FKBP domain binding to alanine peptide mutants. **F** Dissociation constant of SlyD^WT^ FKBP domain binding to charged mutants of peptide residues W4 and F13. The alanine mutants are grey, glutamate mutants red, lysine mutants blue and psWT peptide black. The error bars indicate the error of fit

We have previously established that SlyD possesses a high-affinity substrate binding site in the chaperone domain and a lower-affinity substrate binding site in the PPIase domain (Fig. 1) [14]. To obtain a deeper understanding on how the binding of peptide substrates influences the enzymatic activity of SlyD, we measured the substrate affinities to both SlyD^WT^ and SlyDΔIF constructs by isothermal titration calorimetry (ITC) (Table 1, Fig. 4AB). The dissociation constants (*K*_D_s) were in the expected range for all measured peptide variants, ranging between 0.1–1.1 µM for the chaperone domain and 2–14 µM for the PPIase domain in SlyD^WT^ (Table 1; Fig. 4E, F). In the absence of the chaperone domain (SlyDΔIF construct), the measured affinities to the PPIase domain were reduced (*K*_D_ = 8–52 µM, Fig. 4C, D). In general, the alanine mutations of the peptide substrates led to a slight reduction in their affinities to both SlyD^WT^ and SlyDΔIF (Fig. 4C, E). Similarly, the peptides with positively charged lysine residues at positions 4 (W4K) and 13 (F13K) also displayed only modest changes in their affinities compared to the psWT peptide. On the other hand, the negatively charged glutamate residues at the same positions (W4E, F13E) caused a reduction in their affinity up to fivefold compared to the psWT peptide, further underlining the importance of the distal residues for substrate binding.

**Fig. 5:**
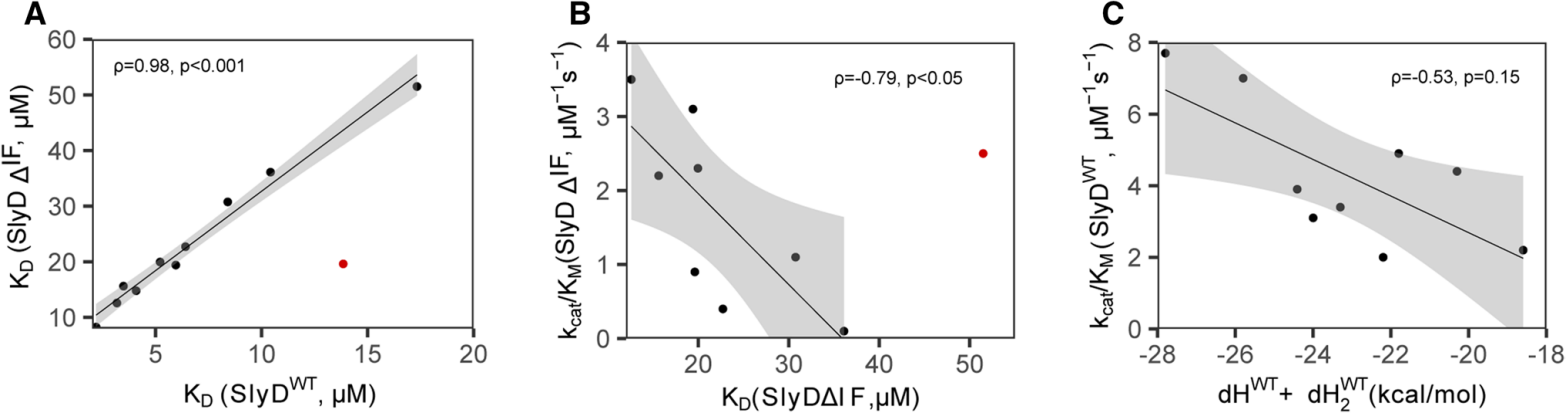
Correlation of binding and activity. The correlations were fitted with a linear model (black line), the grey areas show 95% confidence level intervals- **A** Strong positive correlation between K_D_ of PPIase domain of SlyD^WT^ and SlyDΔIF shows that the chaperone domain increases the binding to PPIase domain approximately by a factor of 3.4. **B** The scatter plot shows that the substrate dissociation constant to the PPIase domain of SlyDΔIF inversely correlates with its activity, indicating that the enzymatic activity depends on the substrate affinity in the absence of the chaperone domain. **C** The scatter plot shows that the correlation between the SlyD^WT^ activity and the sum of enthalpy changes is not significant, which indicates that not only the binding enthalpy, but also other factors play a role in the activity of SlyD^WT^ protein. Spearman`s rank correlation analysis was performed after exclusion of outliers (red dots) by boxplot analysis (see SI Fig. 2)

We have extended these data by measuring the affinities to two additional substrate peptide mutants (G14M, A15L) and used this dataset to explore the relationship between the substrate affinities to the PPIase domain in the presence or absence of the chaperone domain. The bagplot visualization of this relationship depicts a linear correlation between the two (SI Fig. 2A) with only one outlier (W4E). Indeed, the Spearman`s rank correlation test implies a strong correlation (*ρ* = 0.98, *p* < 0.001), indicating that the presence of the chaperone domain increases the affinities of the peptides to the PPIase domain approximately by a factor of 3.4 (Fig. 5A). Our data show that similar to the activity measurements, mutations of residues in the substrate peptide distant from the proline have an impact on their affinity to the PPIase domain of SlyD and the presence of the chaperone domain generally increases this affinity in a linear manner.

### Increased substrate affinity enhances SlyD activity in the absence of the chaperone domain

To explain the changes in the activity of SlyD with different mutant peptide substrates, we correlated the activity data with the binding affinities and the thermodynamic constants derived from the ITC measurements. In case of the SlyDΔIF construct, the bagplot visualization illustrates a linear correlation between substrate affinity (low *K*_D_ values) and enzymatic activity (SI Fig. 2D) with one exception (Y3A). Spearman`s correlation test revealed a negative linear correlation between the substrate dissociation constant and the protein activity (*ρ* = − 0.79, *p* < 0.05, Fig. 5B). Thus, the activity of SlyDΔIF rises with increasing affinity of the peptide substrate to the PPIase domain. In the context of a very efficient enzyme, such correlation can emerge when the variations in the peptide substrate mostly affect the association reaction (*k*_on_).

On the other hand, in the presence of the chaperone domain, such correlation is apparently weak or absent. This is well illustrated by the relationship between the enzymatic activity and affinity of the charged residue mutants at positions 4 and 13. In case of the SlyDΔIF construct, the decreased affinities to peptides W4E and F13E are reflected in reduced enzymatic activities (Figs. 3B vs 4D), however, this behaviour is lost in case of the wild-type protein (Figs. 3D vs 4F). To investigate the relationship between the activity of SlyD^WT^ and the thermodynamic parameters of peptide binding, we performed a principal component analysis (SI Fig. 2F). The analysis confirms that the activity of the SlyD^WT^ indeed does not correlate with the dissociation constant, but rather depends on changes in binding enthalpy and entropy. In particular, the sum of enthalpy changes upon peptide binding to both domains is inversely related to the protein activity (Fig. 5C), however, a linear regression fit does still not yield a significant correlation according to Spearman’s correlation (*ρ* = − 0.53, *p* = 0.15), illustrating that other variables also modulate the activity of SlyD. In summary, our data show that in the absence of the chaperone domain, the activity of SlyD linearly correlates with the dissociation constant of its different peptides, while the presence of the chaperone domain modulates the enzymatic activity such that it does not correlate solely with the affinity to the PPIase domain.

### SlyD structures display conserved substrate binding mode in the PPIase domain

**Fig. 6:**
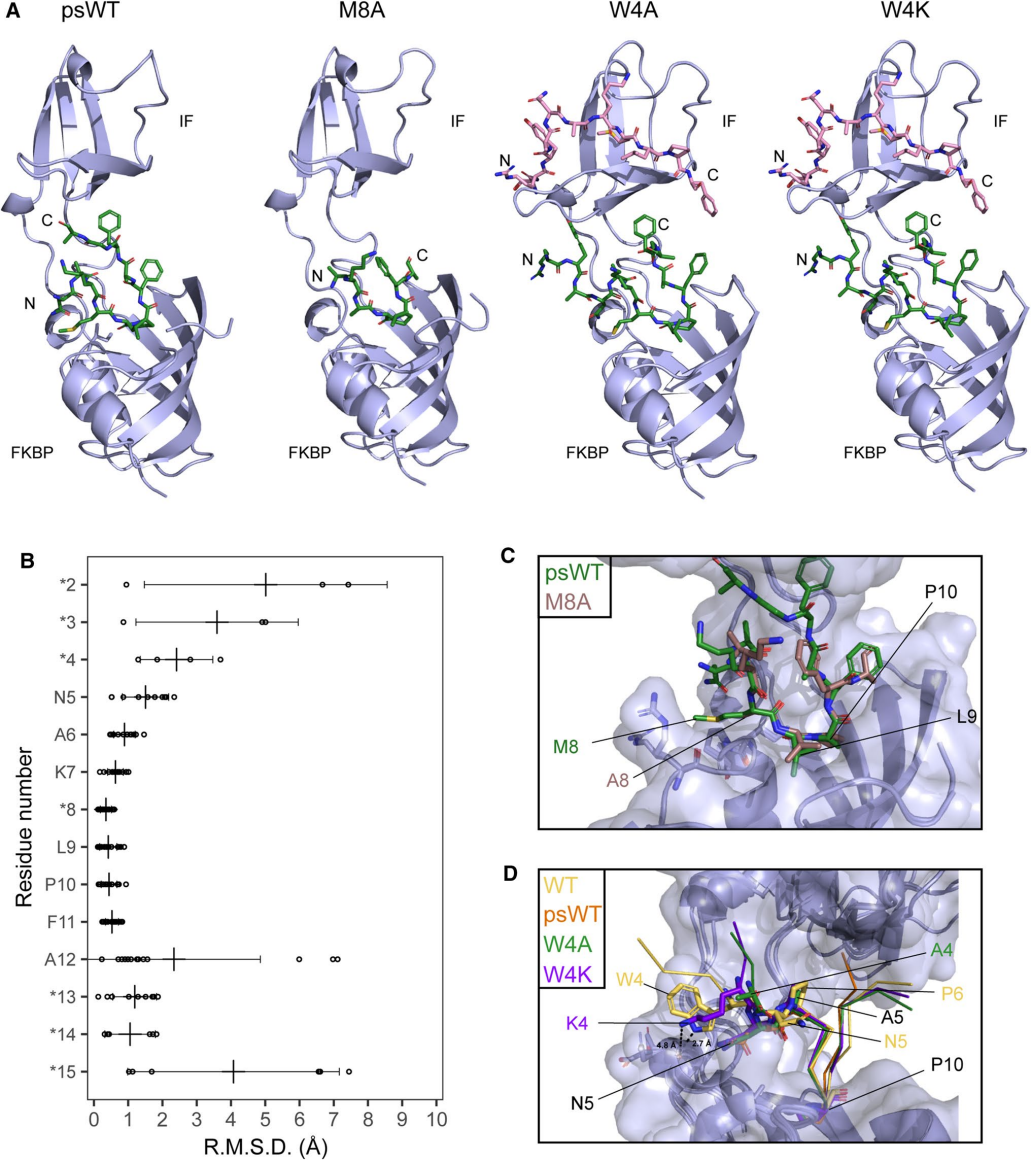
The structural features of substrate binding to SlyD. **A** The solved structures of full-length SlyD with bound substrates in the FKBP domain (psWT, M8A) or in both FKBP and IF domains (W4A, W4K). The structures are aligned by FKBP domain and shown in stereo, FKBP and IF domains are labelled. **B** The chart shows that the position of substrate residues is most conserved in residues 6–11 (*n* − 4 to *n* + 1 of proline residue). Circular points represent RMSDs measured between Cα of each pair of substrate residue, the averages are labelled with the vertical lines and the error bars represent standard deviation. Asterisks signify different peptide residues in the compared structures. **C** Details in the substrate binding in FKBP domain, emphasizing the role of the substrate residue M8. **D** Comparison of FKBP-substrate binding between W4A, W4K and wild-type (WT) substrate [14]. The positioning of the residue 4 is altered in W4A and W4K due to P6A mutation compared to the WT substrate, leading to a lacking interaction with SlyD glutamate 60 (W4-E60 in WT vs K4-E60 in W4K) and different positioning of the N-terminal of the peptide substrate

In order to visualize the binding of the peptide substrates in SlyD, we attempted to co-crystallize SlyD^WT^ in complex with the above-mentioned peptide substrates. Although most of the protein–peptide complexes did not crystallize or yielded crystals of poor diffraction quality, we succeeded in structure determination for four complexes of SlyD^WT^ with bound substrates: psWT, W4A, W4K, and M8A (Fig. 6A). Overall, the resulting models display similar structures as observed previously [14, 19], with some variability in the orientation of the chaperone domain relative to the PPIase domain (Fig. 6A; Table 2). Although all peptide substrates bound to both SlyD^WT^ domains in solution (Fig. 4C–F; Table 1), the chaperone domains displayed no electron density for peptide substrates psWT and M8A in their respective crystal structures (Fig. 6A). The absence of these substrates in the high-affinity chaperone domains can be attributed to high dynamics of substrate binding and serendipitous blocking of the binding site by neighbouring molecule of SlyD in the crystal packing. In all structures, the peptide substrates are bound in the PPIase binding pocket with the proline residue in *cis* conformation, coordinated by the previously observed protein–peptide polar interactions, namely N35-L9, I37-L9, Y63-F11, H119-F11 and Y92-F13 (see SI Table 1). In two complex structures (W4A and W4K peptides, pdb 7OXI and pdb 7OXK), almost all residues of the entire substrate could be modelled (14 out of 15 residues). For peptide M8A, only the central part of the peptide (residues 6–12) was resolved, while for the psWT peptide, clear density for residues 5–15 was visible.

To quantify the position conservation of the substrate in the PPIase binding pocket, we have calculated pairwise Cα RMSD per each residue, comparing the current and previously determined X-ray structures with resolved peptide ligands in the PPIase domain (Fig. 6B). The residues *i* − 5 to *i* + 4 adopt a very similar conformation, while the other residues show high variation in their position in the binding pocket. The specific interactions (*i* − 5 to *i* + 4) can be expected to impact binding and catalysis and therefore these residues probably play a role in both, catalysis and binding, while positions further away mainly form interactions that impact binding.

In our crystal structures, the backbone oxygen of residue *i* − 2, adjacent to the protein active side, forms a hydrogen bond with the hydroxyl group of Y63 of SlyD^WT^ in both cases of psWT peptide (M8) and mutant peptide with methionine (A8). The methionine side chain is partially buried by the loop formed by hydrophobic residues 35–37 of the enzyme. While these additional interactions translate into tighter binding (Fig. 4CE) the overall peptide conformations in the crystal structures are comparable (Fig. 6C). Thus, the M8 side chain of the peptide substrate has an influence on the enzymatic activity of SlyD, which is not caused by a change in the overall binding mode of the peptide. The side chain of M8 might slightly hinder the actual catalytic step or product release (both summed up as *k*_cat_ in the Michaelis–Menten formalism) and thereby decrease the overall enzymatic activity.

On the other hand, the conformational flexibility of the residue *i* − 6 (Fig. 6B) is also reflected in our crystal structures.While residue W4 of the original S2 peptide [14] forms a polar interaction with the SlyD residue E60, orienting the N-terminus of the peptide outside of the binding pocket, mutant peptide residues A4 and K4 do not form this interaction and as a result, the peptide N-terminus bends towards the SlyD linker region (Fig. 6D). Finally, the residue W4 of psWT peptide could not be resolved, indicating flexibility of the peptide N-terminus. Taken together, while different side chains at position 4 assume distinct conformations in each substrate variant, these conformations proof to be unspecific when compared to the structure of other variants. This resembles encounter complexes [46, 47] where initial contacts are unspecific and contribute to the association reaction, but not to the dissociation step where they are replaced by specific contacts. Here, different contacts in the ensemble of different substrates are unspecific and expected to contribute mostly to association of the substrates (*k*_on_) where they contribute directly to enzymatic activity. In general, the observed hydrophobic interactions and hydrogen bonds between the substrate and the protein in the determined structures explain the differences in the measured affinities, but do not correlate with the enzymatic activities of the protein (Table 1), similarly as seen for the measured dissociation constants and enzymatic activities in SlyD^WT^. This supports our observation that the enzymatic activity correlates with *K*_D_ in the absence of the chaperone domain, but in its presence, this effect is overruled and the correlation is lost.

### The chaperone domain ensures specific direction of substrates in the active site

**Fig. 7:**
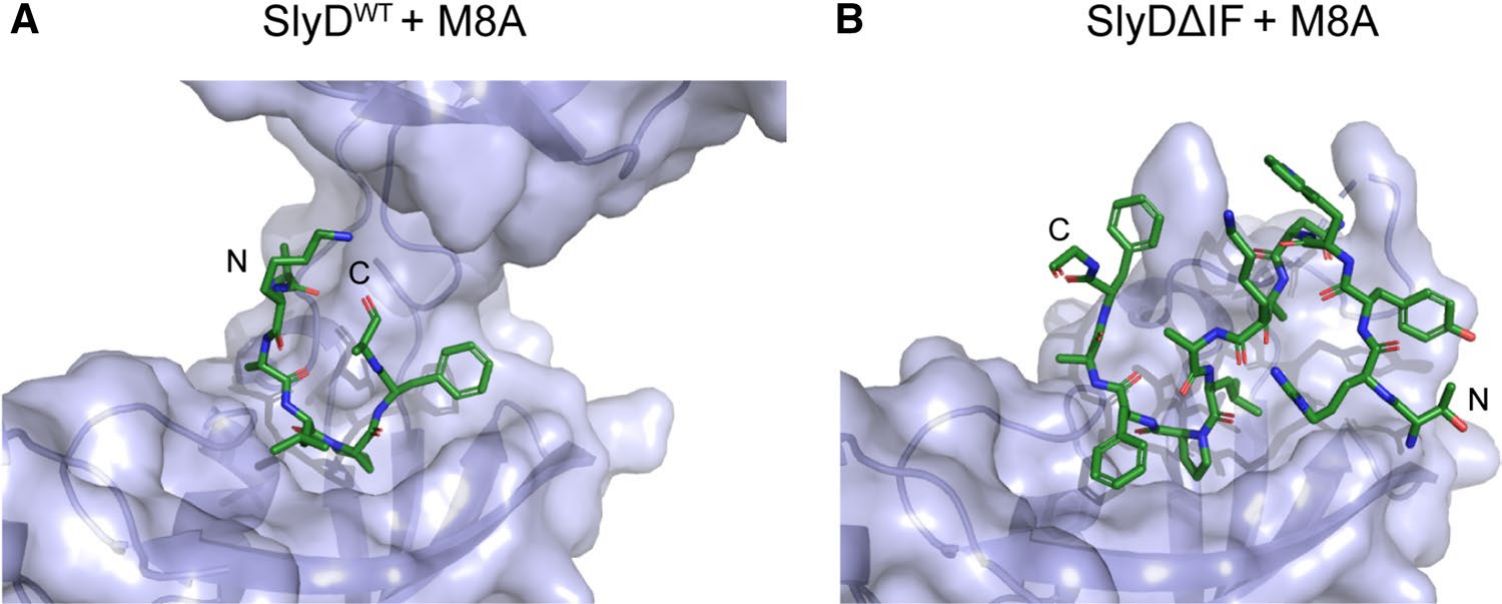
The chaperone domain assures the directionality of the substrate in the PPIase domain. **A** M8A substrate bound in the FKBP domain of SlyD^WT^, displaying the correct orientation. **B** M8A substrate bound to SlyDΔIF, displaying an inverse direction

While the chaperone domain is known to increase the catalytic efficiencies of the enzyme, this cannot be explained by increased binding to the active site alone (SI Fig. 2F). The crystal structure of SlyDΔIF complexed with the M8A substrate offers hints to an additional feature of the chaperone domain that could contribute to increased activity. In this structure, the PPIase domain architecture is well conserved (RMSD of Cα of the core FKBP domain of SlyD^WT^—residues 1–57 and 126–150—and SlyDΔIF is 0.80 Å), but the peptide M8A is bound in an inverse direction (Fig. 7AB). We could model 14 residues of the substrate peptide that displays an opposite directionality compared with SlyD^WT^. However, this peptide binding mode is not possible in case of the peptide M8A in SlyD^WT^ because the bulky side chain of the tryptophan in position 4 of the peptide would sterically clash with the β8-β9 hairpin of the chaperone domain. In our structures described here, we have observed the same substrate bound now in two different orientations in the absence and in the presence of the chaperone domain, implying that the chaperone domain binding provides directionality for substrate binding in the PPIase domain thereby leading to increased activity, by preventing unproductive binding. However, this cannot be the only mechanism by which the chaperone domain increases SlyD activity, as the impact of the chaperone domain differs across our library of substrates (Fig. 3).

### Substrate binding modulates the relative position of SlyD domains

**Fig. 8:**
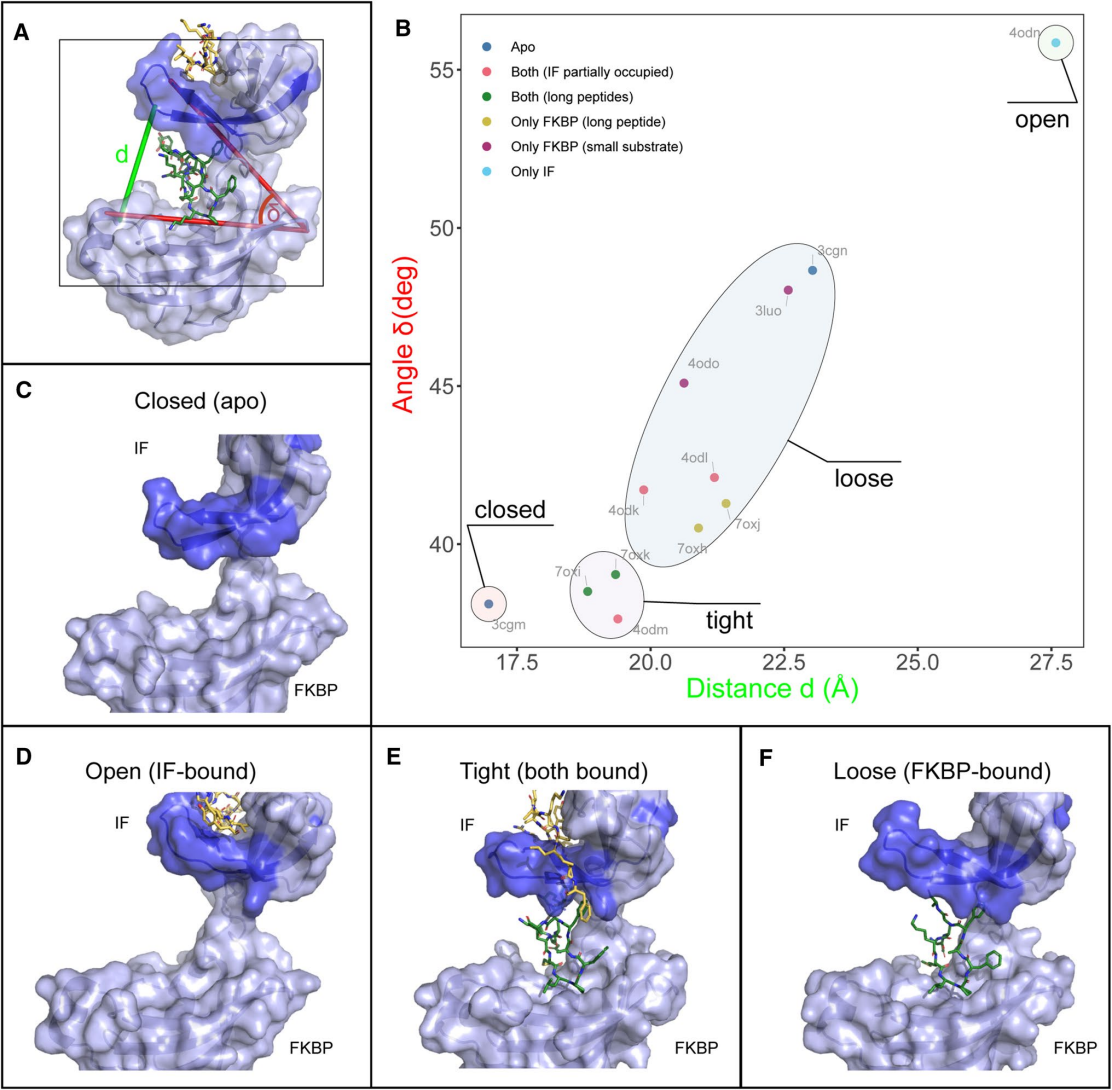
Distinct accessibility to FKBP binding site upon substrate binding. **A** Angle *δ* measured between two SlyD domains (in red) and the distance*d* between the two domains (in green) visualised on the structure of SlyD in complex with ribosomal S2 protein (pdb 4odl). Angle *δ*is defined between residues 145, 19 and 95, while distance *d* is defined between residues 99 (IF domain) and 146 (FKBP domain). **B** Distance *d* plotted against the angle *δ*. The individual points represent the individual structures that are categorised by their ligand binding. The plot divides the structures in four categories based on the similarity in the values of distance *d* and angle *δ*. **C** SlyD in the closed conformation with no substrate bound (pdb 3cgm). **D** SlyD in the open conformation with a substrate bound only in the IF domain (pdb 4odn). **E** SlyD in the loose conformation with a substrate bound only in the FKBP domain (pdb7oxh). **F** SlyD in the tight conformation with substrates bound in both domains (pdb7oxi)

To shed more light on the complex manner in which the chaperone domain influences the PPIase activity, we investigated how peptide binding in the chaperone binding site affects domain orientation and thereby the accessibility of the PPIase active site. As the active site is located between the PPIase domain and the β8–β9 hairpin of the chaperone domain (Fig. 1), the access of longer substrates to this site can be restricted by the proximity and relative position of these two domains and might be regulated by substrate binding in the binding site of the chaperone domain. We therefore used all SlyD structures determined so far as structural snapshots representing the dynamics of SlyD during catalysis and analysed the distances and relative angles between the two SlyD domains in relation to the occupancy of the binding site (Fig. 8AB) The visual inspection of the structures supported by derived parameters (the distance *d* and the angle *δ* between the IF and FKBP domains, see Fig. 8A) enabled us to classify the structures in four groups with distinct conformations: closed, tight, loose and open (Fig. 8B–F). The closed structure is represented by a single SlyD^WT^ structure in the absence of substrates (pdb 3cgm [19]). In this conformation, the PPIase and chaperone domains are in close proximity and the access to the PPIase binding site is limited (Fig. 8C). Upon peptide binding to the high-affinity site in the chaperone domain, the distance between the binding site and the chaperone domain increases as SlyD switches to an open conformation, facilitating easier access to the active site of the isomerase (Fig. 8D). When both binding sites are fully occupied, the two domains restrict access to the PPIase domain binding site in the tight conformation (Fig. 8E). Here, the release of the substrate from the FKBP binding site is likely hindered. Finally, the loose conformation is defined by the newly determined structures with the substrate bound only in the FKBP domain. In these structures, the access to the FKBP binding site is not restricted and the peptides bound in the FKBP domain can freely diffuse from the binding site upon its release (Fig. 8F). In addition, another apo-structure of SlyD^WT^ (pdb 3cgn [19]) exhibits the loose conformation, indicating that the apo-protein is dynamic and adopts different conformations in solution. In some of the previously determined structures, it has also been seen that only a portion of the binding site of the chaperone domain is occupied by the peptide (pdb 4odk, 4odl, 4odm), while the substrate is also bound in the PPIase active site. In these cases, the relative orientation of the domains is placed between the loose and tight conformation, illustrating that the entire length of the substrate is required to be bound in the chaperone domain to stabilize the tight conformation (Fig. 8B). Of note, in the SlyD^WT^ structures with a short tetrapeptide (pdb 3luo [19]) or the molecule FK506 (pdb 4odo [14]) bound in the PPIase domain, the SlyD^WT^ conformations falls into the loose conformation category. However, the distance between the SlyD^WT^ domains is increased in the presence of these small substrates compared to structures with long peptides, offering another explanation to why short peptides are catalysed less efficiently compared to longer peptides (Fig. 8B). Our analysis highlights that the chaperone domain does not only function as a holdase to increase the local concentration of the substrate in the vicinity of the PPIase domain [48], but also undergoes conformational changes upon peptide binding that could result in allosteric modulation of the isomerase activity. Such allosteric modulation would be achieved by the alteration of substrate access to the active site as the enzyme switches between the individual conformations, during a catalytic cycle depending on the occupancy of both domains by their substrates.

## Discussion

### Remote substrate residues have impact on PPIase activity

In this work, we can show that SlyD PPIase activity does depend on proline-distant residues (Fig. 3) of the substrate peptide, using a new NMR-based method for larger peptide substrates. The influence of the residue *i* − 1 and *i* + 1 from proline has been studied previously [11, 33]. Our data illustrate that residues up to *i* − 8 and *i* + 3 can have a significant impact on enzymatic activity. RMSD values of all peptides bound to the active site in crystal structures revealed that residues *i* − 5 to *i* + 3 of the proline adopt a similar conformation, which might represent the structural requirement for proline isomerization. Therefore, certain side-chains outside the region of conformational restriction impact activity and binding strength. Mutating the previously observed interacting aromatic residues (*i* − 6 and *i* + 3), to alanine led to a reduced activity and affinity, whereas positively charged lysine residues did not cause any activity and affinity change in SlyDΔIF. The importance of aromatic and positively charged residues of the substrate can be interpreted as forming initial contacts during association with in the mostly hydrophobic and negatively charged binding pocket of the PPIase domain. These interactions are presumably not needed for the actual catalytic step, since they located outside the structural core region. In SlyDΔIF the substrate dissociation constants inversely correlate with the protein activity (Fig. 5). For a superefficient enzyme like SlyD with high enzymatic efficiency (*k*_cat_*/K*_M_ > 10^6^ M^−1^ s^−1^) [12, 14, 20, 32] and a high catalytic turnover (*k*_cat_ > 10^5^ s^−1^) [14] for unfolded peptide substrates changes in enzymatic activity (*k*_cat_/*K*_M_) correspond to changes in *k*_on_, as the process is limited by association. A decrease in *K*_D_ (*k*_off_/*k*_on_) should result in an equal increase in *k*_cat_/*K*_M_ only if it arises from a changed *k*_*on*_. The above-described role of remote aromatic and positively charged side chains nicely fits to this. They engage in initial unspecific contacts which are not decisive for a distinct conformation, but simply increase the association of the substrate and consequently the enzymatic activity.

### Interactions increasing the stability of the enzyme substrate complex have a slightly negative impact on activity

An interesting exception to the coupling of the substrate affinity and protein activity is the mutation M8A that slightly reduces the peptide binding affinity but significantly increases the protein activity (Table 1). We hypothesize that such inverse effect can be explained by the proximity of the methionine residue (*i* − 2) to the proline. Although the interactions of the proline-distant residues are beneficial for SlyD activity because they simply attach the substrate to the PPIase binding pocket (*k*_on_), the interaction of the proximal residues are detrimental, because such interactions in the proximity of the proline residue hinders the transition from *cis* to *trans* conformation or product release (*k*_cat_). The mutation of methionine 8 to alanine removes the hydrophobic interactions mediated by the methionine side chain, as is visible in the crystal structure (Fig. 4B) and thus facilitates proline isomerization.

### Complex impact of the chaperone domain on PPIase activity

In the presence of the chaperone domain, the binding affinity of the PPIase domain generally increases by a factor of 3.4. However, the correlation between the binding affinity and protein activity is no longer observed, indicating that the impact of the chaperone domain on the activity of SlyD is rather complex. In particular, the chaperone domain does not simply increase the association of the substrate. Indeed, our PCA analysis showed that SlyD^WT^ activity is influenced rather by the binding enthalpy of both domains, and not just simply by the substrate binding affinity (enthalpy and entropy). However, the correlation between the enzymatic activity and the sum of binding enthalpies is not significant, indicating that other variables likely contribute to the differences in the measured thermodynamic constant.

So far, the chaperone domain has been thought to increase the SlyD activity by its high dynamics, binding the substrates with a higher affinity and thereby increasing the local concentration for PPIase binding [48] after releasing it. However kinetic considerations argue against such a mechanism. Assuming a diffusion limited association constant of *k*_on_ = 10^8^ M^−1^ s^−1^ and dissociation constants (*K*_D_) for the chaperone domain of < 1 µM, one can estimate the dissociation rate of the chaperone domain *k*_off_ to 100 s^−1^ or less, which is far below determined *k*_cat_ values of (740 000 ± 140 000) s^−1^ [14].

### Allostery in the catalytic cycle of SlyD

The substantial number of available crystal structures of SlyD^WT^ in complex with different substrates of different occupancy in both binding clefts allows a more rigorous structural analysis. Our analysis highlights that substrate binding alters the relative position of the chaperone domain, resulting in a movement of the β8–β9 hairpin between distinct conformational states, thereby regulating access to the PPIase active site (Fig. 8). In our analysis, the two apo structures of SlyD fall into the closed and loose conformational states, which is in agreement with single molecule observations [22]. However, the structure of substrate bound only to the chaperone domain represents an open state with a larger distance between the two SlyD domains and far more accessible active site. In comparison, binding of an additional substrate in the PPIase binding site (both sides occupied) results in a tight conformation, in which the release of the substrate from the PPIase binding site is hindered. Finally, our structures presented here displaying the substrate bound only in the PPIase binding site but with an unoccupied chaperon binding site, assume a loose conformation, in which the release of the substrate is possible. Allosteric coupling is further supported by comparing differences in the reported activities using SlyD with and without the chaperone domain, where activity measurements were performed at different substrate concentration and with substrate that display different affinities to SlyD. In studies with low affinity tetrapeptides at concentrations below 100 µM, no positive impact of the chaperone domain was observed [11, 12], while at 500 µM tetrapeptide concentration activity was increased by a factor of 1.7 in the presence of the chaperone domain [14]. In this study, using 15-residue long peptides with higher affinity at concentrations of 100 µM, in which the chaperone domain is occupied, we observe an increase of a factor of 2.2 by the chaperone domain in case of the pWT substrate. For partially folded protein substrates, this factor further increases to 200 [11, 12]. This here proposed allosteric model is as good as an explanation for the impact of the chaperone domain as previous models that proposed an increase of the local substrate concentration by binding and releasing substrate to the chaperone domain, but does not run into conflicts between *k*_off_ and *k*_cat_ as discussed above.

**Fig. 9:**
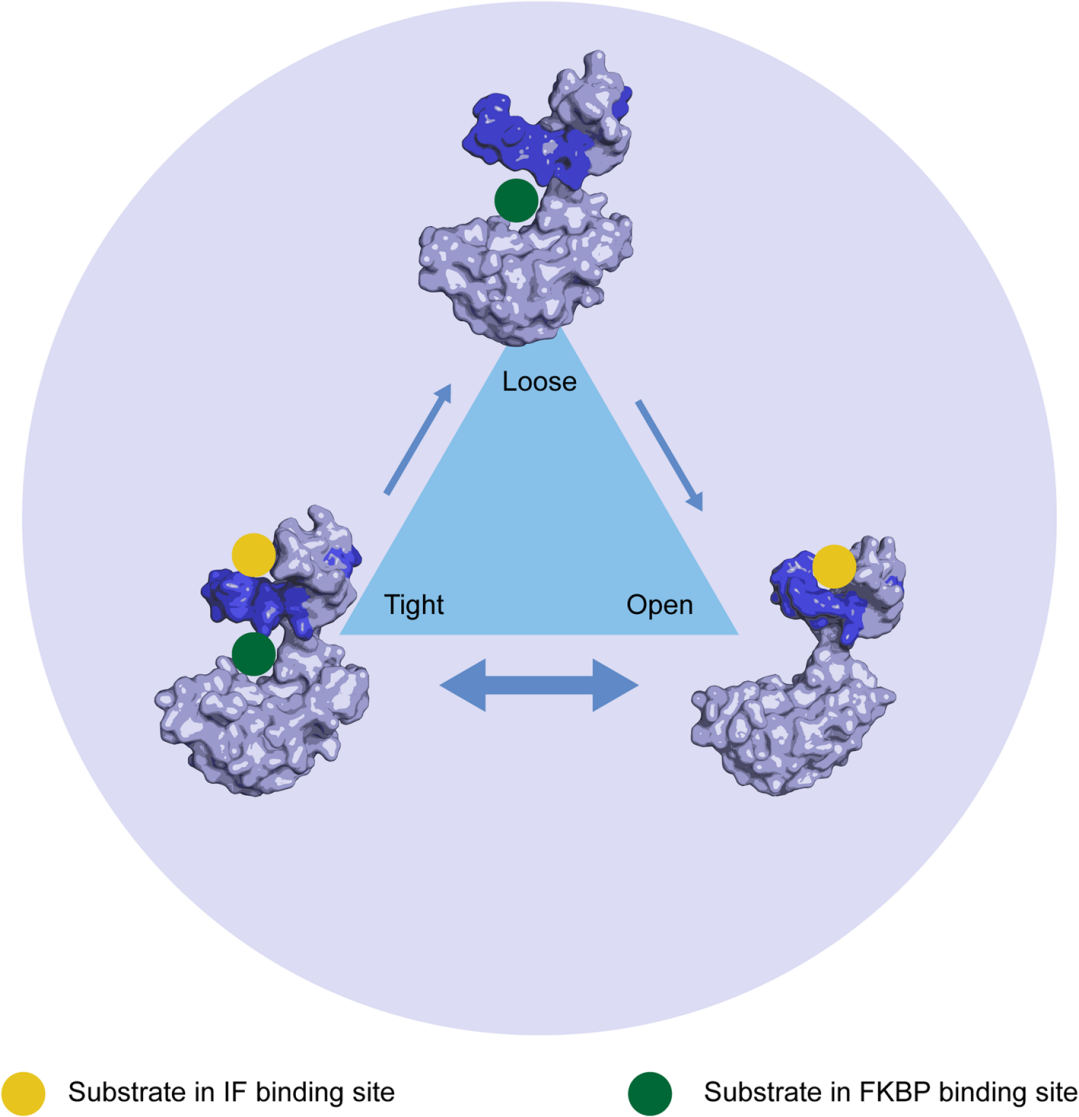
A model of allosteric regulation of SlyD. The combination of substrates bound in the chaperone (IF) domain and the PPIase (FKBP) domain modulates the relative orientation of the two domains. When only the PPIase domain is occupied by the substrate, SlyD assumes an open conformation with good accessibility to the PPIase active site. When both peptides are bound, SlyD adopts a tight conformation, during which the *cis–trans* isomerization is catalysed and the release of the substrate peptide is restricted. When only the PPIase domain is occupied by a substrate, SlyD displays a loose conformation, in which the access to the PPIase domain is not restricted anymore

This global analysis allows us to propose a model of allosteric regulation of SlyD (Fig. 9) that is compatible with domain interplay and the previously suggested increase of the local substrate concentration by the chaperone domain. We hypothesize that the peptide substrate binding in the high-affinity chaperone domain is stabilizing the open conformation of the PPIase domain and enabling the subsequent binding of another substrate in the PPIase domain. The binding of the second substrate induces the tight conformation, in which the substrate release from the PPIase domain is restricted. We assume that the proline isomerization occurs at this step, as all known structures of SlyD and its analogues possess a proline in the *cis* conformation, which is likely the result of the enzymatic catalysis. Energetically the less populated and thus energetically disfavoured *cis* conformation is closer to the transition state compared to the *trans* conformation. Preferential binding of the *cis* conformation is the structural consequence of this. Finally, the peptide can be released from the PPIase binding site, triggering a conformational change back to an open state with a substrate bound in the chaperone domain only. Alternatively, the substrate bound in the chaperone domain can dissociate first, switching SlyD to the loose conformation that enables easier release of the second substrate from the PPIase. In this alternative scenario, the substrate released from the chaperone domain can subsequently bind to the PPIase domain, maintaining its orientation given by the chaperone domain.

## Conclusions

Residues remote from the proline up to *n* − 8 and n + 3 have an impact on peptidyl-propyl isomerase activity of SlyD and its substrate binding, although some of them are outside the conformationally conserved core region of the substrate bound in the active site. We hypothesize that they engage in the association reaction but not form distinct interactions in the bound state. We observed that in the absence of the chaperone domain, the protein activity linearly correlates with the affinity of the substrates, whereas in the presence of the chaperone domain additional factors modulate the protein activity. A key factor is allosteric communication between the chaperone and PPIase domain. Their relative orientation altering the accessibility of the PPIase active site, is modulated by the presence of substrates bound to both chaperone and PPIase domain.

## Supplementary Information

Below is the link to the electronic supplementary material.

Supplementary file1 (DOCX 537 KB)

Supplementary file2 (XLSX 48 KB)

Supplementary file3 (XLSX 12 KB)

## Data Availability

All data generated or analysed during this study are included in this published article and its supplementary information files.
