# Supplementary material for: Impact of distant peptide substrate residues on enzymatic activity of SlyD
Source: Cell Mol Life Sci. 2022 Feb 19;79(3):138. doi: 10.1007/s00018-022-04179-4 (PMC8858294; doi:10.1007/s00018-022-04179-4)
Supplement: Supplementary file 1 — Supplementary file1 (DOCX 537 KB) [file 18_2022_4179_MOESM1_ESM.docx]

**
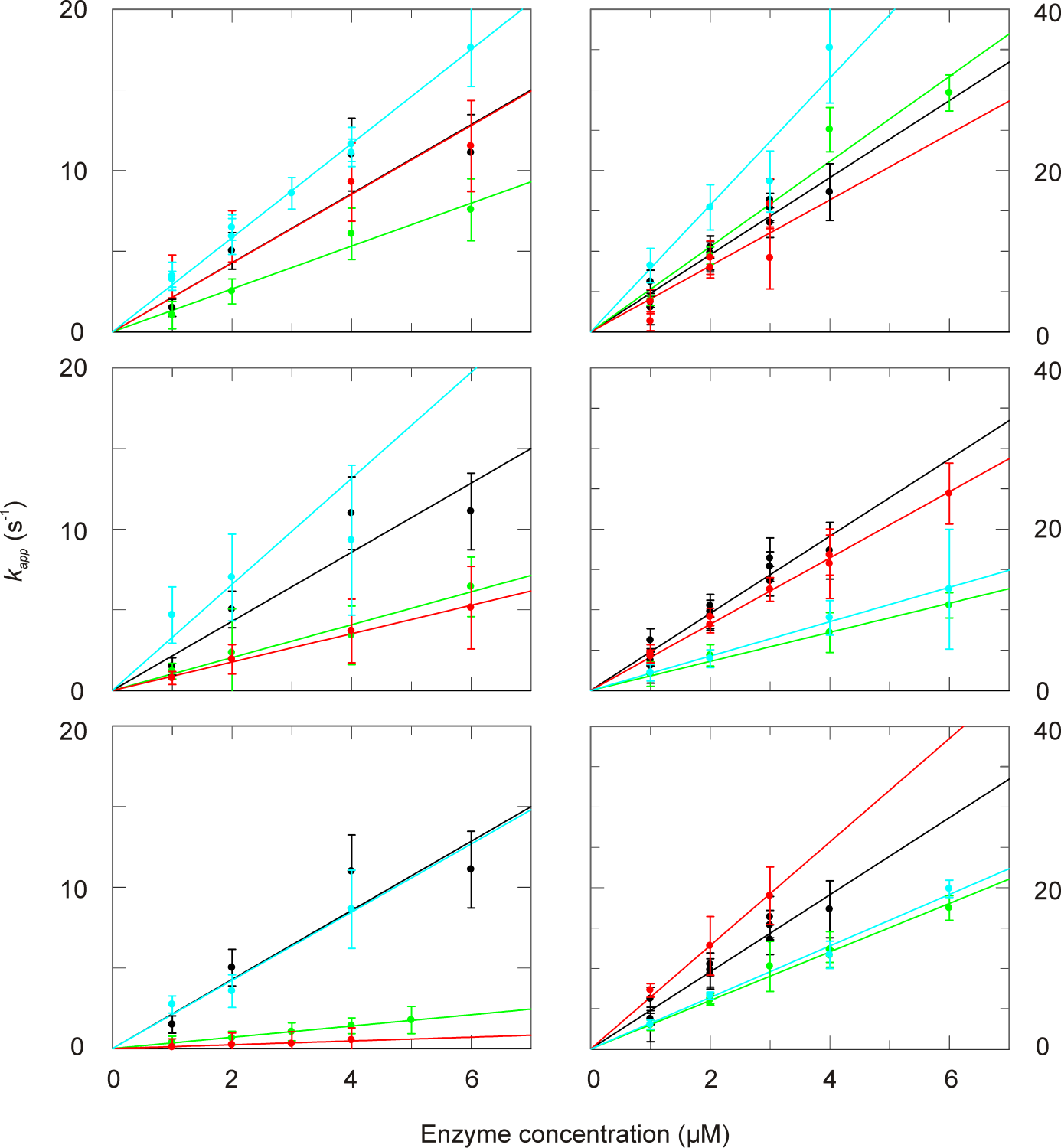
**

**SI Fig. 1:** Derived exchange rates plotted against enzyme concentration and fitted linearly in order to derive *k*_cat_*/K*_M_ of SlyDΔIF (left column) and SlyD^WT^ (right column). Up: psWT (black), R2A (green), Y3A (red) and M8A (blue). Middle: psWT (black), W4A (green), W4E (red) and W4K (blue). Down: psWT (black), F13A (green), F13E (red) and F13K (blue).


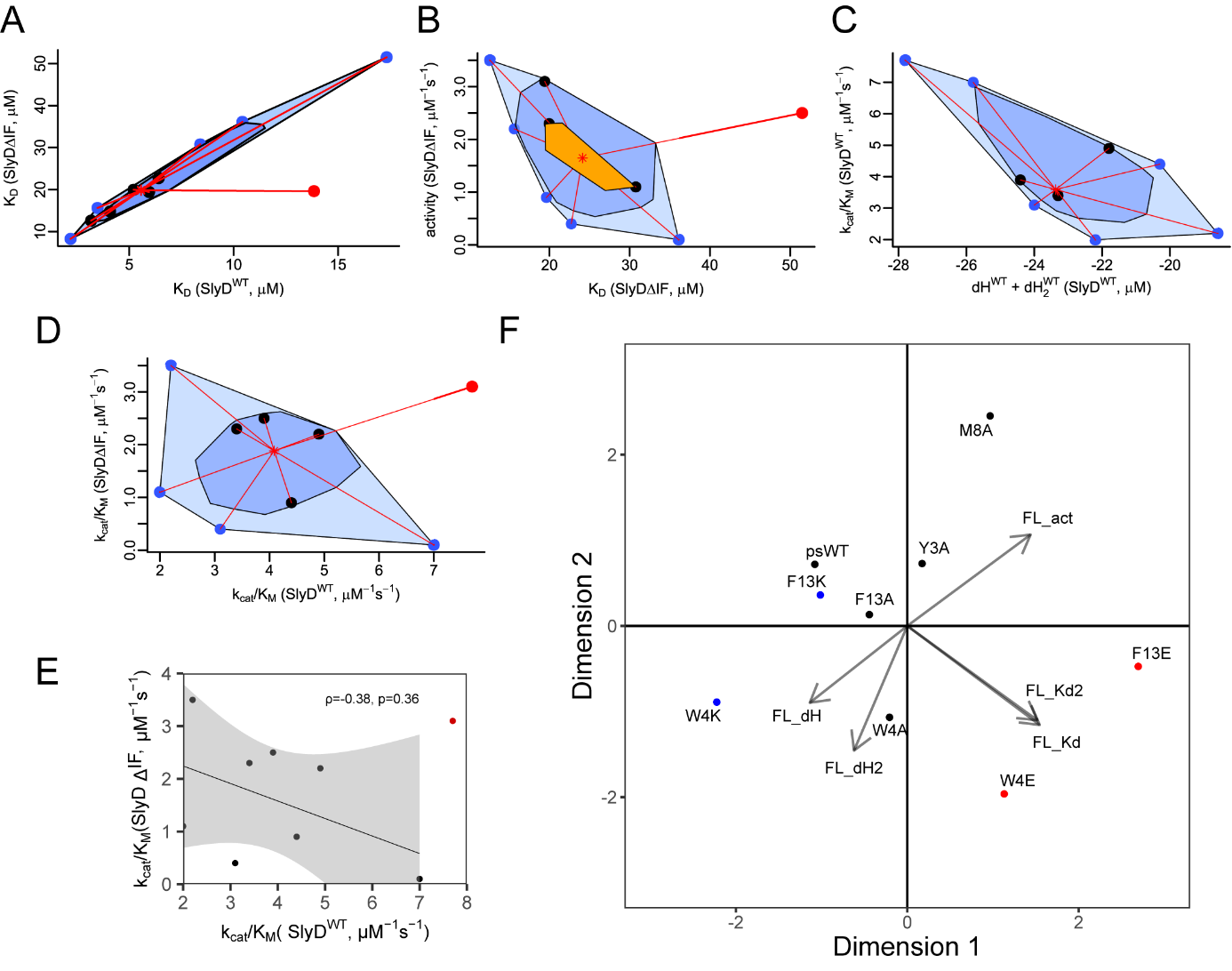


**SI Fig. 2:** (A-D) The bagplots were used to identify the outliers prior to the correlation analysis. Half of the observations is included in the bag (dark blue background), whereas the rest of the observation are included in the fence (light blue background). The outliers outside of the fence are red. (A) Bagplot implies that the K_D_s of the PPIase domain of SlyD^WT^ and SlyDΔIF linearly correlate and identifies one outlier (W4E). (B) The bagplot implies a linear correlation between SlyDΔIF substrate affinity and activity and identifies one outlier (Y3A). (C) Bagplot of the sum of binding enthalpies of SlyD^WT^ binding sites plotted against the catalytic efficiency of SlyD^WT^. (D) Bagplot of catalytic efficiency (*k_cat_/K_M_*) of SlyD vs SlyDΔIF with one outlier (M8A).(E) The correlation analysis of SlyD activities in the presence (SlyD^WT^) and absence (SlyDΔIF) of IF domain. (F) The principal component analysis suggests that the activity of SlyD^WT^ is not dependent on the K_D_ of substrate but on their binding enthalpy, stressing the importance of the IF domain.
